# Simultaneous Quantitative Detection of Six Families of Antibiotics in Honey Using A Biochip Multi-Array Technology

**DOI:** 10.3390/vetsci6010001

**Published:** 2018-12-28

**Authors:** Roberta Barrasso, Elisabetta Bonerba, Alessandra Emilia Savarino, Edmondo Ceci, Giancarlo Bozzo, Giuseppina Tantillo

**Affiliations:** Department of Veterinary Medicine, University of Bari Aldo Moro, strada Provinciale per Casamassima km 3, 70010 Valenzano (BA), Italy; elisabetta.bonerba@uniba.it (E.B.); savarino.alessandra@virgilio.it (A.E.S.); edmondo.ceci@uniba.it (E.C.); giancarlo.bozzo@uniba.it (G.B.); giuseppina.tantillo@uniba.it (G.T.)

**Keywords:** antibiotic residues, biochip, honey, multi-array

## Abstract

Chemical residues of veterinary drugs such as streptomycin, chloramphenicol, macrolides, sulphonamides, tetracyclines, quinolones and aminoglycosides and other contaminants such as pesticides and heavy metals have been found in honey, leading to concerns for human health. Indeed, there is a growing interest in their presence and persistence in the environment because low levels of antibiotics may favour the proliferation of antibiotic-resistant bacteria. Moreover, antibiotics present in honey may produce residues in foodstuffs, causing adverse effects on humans such as allergic reactions, toxic effects and damage to the central nervous systems. For food and health/safety reasons, antibiotic drugs are not authorized for the treatment of honey bees in the EU, even though these antimicrobial drugs have been approved in many third-party countries. For this reason, contaminated honey products can still be found in European markets. Therefore, there is a need to develop a precise, accurate and sensitive analytical method that may be used to simply and rapidly detect these compounds in honey. The aim of our study was to detect the presence of antibiotics in Apulian honey using the Anti-Microbial array II (AM II) as an innovative screening method to test the health quality of honey and honey products.

## 1. Introduction

The presence of antibiotics in honey or in any other food is problematic because they may produce residues in foodstuffs and present risks to human health, for example, by causing allergic reactions, antibiotic resistance in humans [1], toxic effects and damage to the central nervous system [2,3]. For these reasons, the use of antimicrobial compounds in food-producing animals has been banned or restricted by many countries, and no market authorisation may be obtained without Maximum Residue Limits (MRLs) [4]. Commission Regulation (EU) No. 37/2010 [5] established MRLs for pharmacologically active substances in foodstuffs of animal origin. The annex of this regulation shows the list of allowed substances, including those concerning honey, which has MRLs that have been set for Amitraz (MRL 200 μg/kg) and Coumaphos (MRL 100 μg/kg). On the other hand, the pharmacologically active substances in honey for which MRLs have not been indicated currently include Formic Acid, Lactic Acid, Oxalic Acid, Camphor, Eucalyptol, Flumethrin, Menthol, Tau fluvalinate and Thymol.

In 2012, it was decided to restrict zero-tolerance to residues of non-allowed substances while residues of allowed substances should be judged based on scientific risk assessment [6]. The regulatory limit for certain prohibited or unauthorized analytes in food of animal origin is the Minimum Required Performance Limit (MRPL), defined as the minimum content of an analyte in a sample, which must be detected and confirmed by laboratories.

In Italy, the National Residues Control Plan (NRCP) recently implemented detection limits for antibacterial substances in honey as 5 μg/kg for sulfamides, tetracyclines and macrolides and 1.3–1.6 μg/kg for aminoglycosides. These concentrations of antibacterial substances or limits of detection represent the minimum levels that laboratories have to guarantee for control activities [7]. For substances without MRLs, the Community Reference Laboratories (CRLs) distributed a guidance paper providing recommended concentrations in order to improve and harmonize the performance of analytical methods for national residue control plans [8].

For food and health/safety reasons, antibiotic drugs were not authorized for the treatment of honey bees in the EU, even though these antimicrobial drugs have been approved in many third-party countries [9]. As a result, due to the high import quota from other countries, contaminated honey products may be found in European markets [10]. Given that this undermined the image of bee products as natural and healthy [11], the importation of honey from China, the world’s largest producer, was banned in 2002–2004. Nowadays it is still barely accepted due to antibiotics being found in several honey samples [12]. Antibiotic detection led to the withdrawal of the corresponding batch and had a serious impact on the reputation and the economy of the producer and its country, as well as that of the European distributor [13].

The broad activity spectrum of antimicrobials led to their widespread use in veterinary practice from the 1950s onwards, contributing to the onset of bacterial resistance. Hence, new synthetic agents, e.g., the class of quinolones (Qnl), were developed to replace ineffective antibiotics [14]. Indeed, the administration of the Qnls oxolinic acid, flumequine, marbofloxacin and enrofloxacin has been proposed to cure and prevent bacterial or other bee diseases, to reduce the loss of honeybee populations as well as prophylactic agents [15,16]. In fact, ceftiofur and thiamphenicol are not widely used for bee treatment; on the contrary, tetracycline, tylosin, streptomycin/dihydrostreptomycin and quinolones are used extensively and should be screened in honey. In recent years, these antibiotics have been used in apiculture to treat bacterial brood infections and although not intended for use during the production of marketable honey, their residues can persist in honey (Investigator™ EV 3524). Moreover, bee colonies are susceptible to several types of infestations and disease including varroosis and American and European foulbrood [17] and the impact of these represents a global threat to apiculture [18].

According to Chiesa et al. (2018) [19], the sources of honey contamination may be divided into environmental and apicultural (antibiotics are included in the latter). The detection of antibiotic residues in honeys, regardless of the production area, could provide interesting evidence of the close relationship between the presence of antibiotics and beekeeping practices. Therefore, there is a need to develop a precise, accurate and sensitive analytical screening method that may be used to easily and quickly detect antibiotics in honey. The aim of our study was to reveal the presence of antibiotics in Apulian honey using the Anti-Microbial array II (AM II) method, a competitive chemiluminescent assay. Biochip array technology is a multi-analyte testing platform which allows for the simultaneous quantitative or qualitative detection of a wide range of analytes from a single sample. It provides a unique platform for assessment of honey samples in a rapid, accurate and easy to use format.

## 2. Materials and Methods

### 2.1. Sampling

Samples of honey were selected to obtain groups of samples from different botanical and geographical origins: sixty-six honey samples of nine floral origins were used, these being multi-flower (18), cherry (16), citrus fruit (15), honeydew (4), coriander (4), thyme (3) acacia (3), centaury (2) and almond (1). These last two botanical varieties were referred to as “other”. Regarding the territorial origin, the samples came from the provinces of Bari, Foggia and Taranto and were provided by the same beekeepers. The honey samples were divided into two groups, based on the year of production. The first group, consisting of twenty-four samples of Apulian honey, was produced in 2016 while the second, consisting of forty-two samples, was made up of Apulian honey produced during 2017. Specifically, the botanical origins of the honey samples of the first group were multi-flower (3), cherry (12), citrus fruit (8) and coriander (1); on the other hand, the floral origins of the honey samples of the second group were multi-flower (15), cherry (4), citrus fruit (7), honeydew (4), coriander (3), thyme (3), acacia (3) and other (3).

Quantitative detection of multiple analytes was determined as described in a previous study [9] although, this was not confirmed using AOAC (Association of Analytical Communities) based methods. Briefly, the concentration of the analytes in the sample was found by the Evidence Investigator™ Anti-Microbial Array II (Investigator™ EV 3524; Randox Laboratories Limited, Crumlin, County Antrim, UK), and was obtained by following the manufacturer’s guidelines. The kit used in this work is CE marked for veterinary use and/or for the analysis of food only and the Evidence Investigator™ is a trademark of Randox Laboratories Ltd. (Crumlin-Aldergrove, Antrim Northern Ireland). The manufacturer has sole and ultimate responsibility for the conformity of the product to the applicable assay.

### 2.2. Principle

The Evidence Investigator™ Anti-Microbial Array II is a commercially available kit that quantitatively tested for quinolones, ceftiofur, thiamphenicol, streptomycin, tylosin and tetracyclines, simultaneously. Each kit consisted of six carriers, nine points of calibration, buffers required to reconstitute and other reagents (conjugate, chemiluminescent solution). Each carrier was composed of nine microarrays (1 × 1 cm). Then, using a kit, it was possible to analyse a calibration carrier and five carriers of sample (45 samples). A carrier may be divided into three × three biochips, consequently, a number of samples multiplied by of three could be investigated.

The Evidence Investigator™ Anti-Microbial Array II was used to perform simultaneous quantitative detection of multiple analytes from a single sample. The core technology was the Randox biochip, a solid substrate containing an array of discrete test regions of immobilised antibodies specific to different antimicrobials. A competitive chemiluminescent immunoassay was employed. Increased levels of antimicrobial in a sample led to decreased binding of antimicrobial labelled with horseradish peroxidase (HRP) and thus a decrease in the chemiluminescence signal that was emitted. Indeed, the biochip detection system is based on a chemiluminescent signal. This is the emission of light, without heat, as a result of a chemical reaction. An enzyme is used to catalyse the chemical reaction on the biochip, which generates the chemiluminescent signal. The light emitted from the chemiluminescent reaction that takes place in each discrete test region (DTR) is simultaneously detected and quantified using a Charge-Coupled Device (CCD) Camera at −50 °C. This CCD Camera simultaneously records the light emission from all the discrete test sites on each biochip on the biochip carrier. The concentration of the sample analytes was then calculated from the stored calibration curve. Kit components were lot-specific and barcode-matched. Components from different lots had to be kept apart.

### 2.3. Honey Procedure for Sample Preparation

A total of 1 g of honey sample was used. Then 9 mL of diluted wash buffer (containing detergent and preservatives) warmed to 37 °C was added. The solvent-resistant tubes (Falcon™ Round-Bottom Polypropylene Tubes, Fisher Scientific Company, Ottawa, Ontario) were placed on a roller for 10 min or until dissolved. The preparation was diluted with an equal amount of diluted wash buffer, e.g., 1 mL + 1 mL. The sample was now ready to be applied to the biochip. The Evidence Investigator^TM^ analyser did not automatically account for sample dilution. The concentration reported was manually multiplied by 20 to obtain the final sample concentration. For the honey protocol, the calibrators and the control were reconstituted with the reconstitution buffer provided.

### 2.4. Calibration and Materials

A nine-point calibration was performed using the AM II calibrators, which covers the measuring range of all assays. A maximum of 6 biochip carriers may be assayed simultaneously, and it was recommended that a new calibration curve be constructed for each assay series. All materials were equilibrated to room temperature prior to use. It was recommended to: (i) calibrate the first carrier to be assayed using the nine calibrators provided; (ii) equilibrate the thermo-shaker at 25 °C for 30 min prior to use; and (iii) perform this assay in a laboratory with a temperature between 15 and 25 °C. Operating in a laboratory outside these temperatures could have an adverse effect on the performance.

### 2.5. Assay Protocol

For the Evidence Investigator™ AM II assay, the maximum number of carriers assessed at any one time was dependent on reagent/sample loading time and it was recommended that this period should not exceed 10 min. The addition of reagents was done by pipetting towards the back edge of the biochip, taking care not to touch the biochip surface with the pipette tip. Assay reagents and samples were added with the tip of the pipette pointing towards the back of the biochip. The procedure was as follows.

(i) All samples and reagents were prepared as described in previous sections. (ii) 100 μL of assay diluent solution (ready for use) was pipetted into the appropriate biochip wells as required. This was a buffer at pH 7.2 containing protein, detergent, blocking agents and preservatives. (iii) 100 μL of calibrator or sample was pipetted into the appropriate biochip wells. The calibrator consisted of 9 vials of lyophilised base material containing analytes for the full panel. All edges of the handling tray were gently tapped in order to mix the reagents. (iv) The handling tray was secured to the base plate of the thermo-shaker and it was incubated for 30 min at 25 °C and 370 rpm. (v) 100 μL of the working strength conjugate was pipetted into the appropriate biochip wells and the tray was incubated for a further 60 min at 25 °C and 370 rpm. (vi) The handling tray containing the carriers was removed from the thermo-shaker. The reagents to waste were discarded by sharply flicking the handling tray. (vii) Two quick wash cycles were immediately carried out, using approximately 350 μL of wash buffer for each biochip well. The handling tray was gently tapped to release any reagents trapped below the biochip. Care needed to be taken not to overfill the biochip wells during washing, so as to reduce the potential for well-to-well contamination. A further four wash cycles were carried out. For each cycle, all four edges of the handling tray were gently tapped for approximately 10-15 s; then the biochips were left to soak in wash buffer for 2 min. (viii) After the final wash, the wells were filled with wash buffer and left to soak until image acquisition. No carrier was left to soak for longer than 30 min.

The carriers were processed individually and those awaiting imaging were protected from light. The first carrier was removed from the handling tray to be imaged. Just before the addition of the signal, the wash buffer was removed using a sharp, flicking action and the carrier was tapped onto lint free tissue to remove any residual wash buffer. 250 μL of working signal reagent-EV805 was added to each biochip well and they were covered to protect them from the light. The working signal reagent-EV805 was composed of Luminol-EV805 (1 × 10 mL) and Peroxide (1 × 10 mL) mixed in a ratio of 1:1. After exactly 2 min (±10 s), the carrier was placed into the Evidence Investigator™. The use of a timer was recommended to ensure imaging was carried out at the correct time. The captured images were automatically obtained as defined by a dedicated software application and the results were automatically processed using an integrated graphical user interface and e-touch software.

### 2.6. Sensitivity

The limits of detection (LOD) for the Evidence Investigator™ AM II analytes were determined in several food matrices. The LOD for the honey is shown in Table 1.

## 3. Results

**Figure 1:**
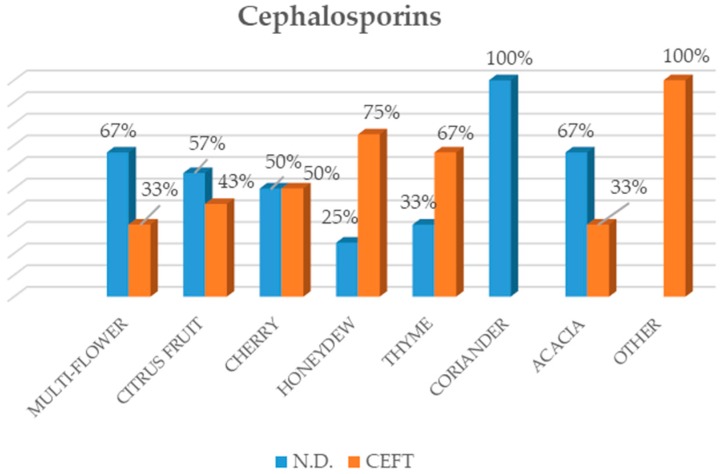

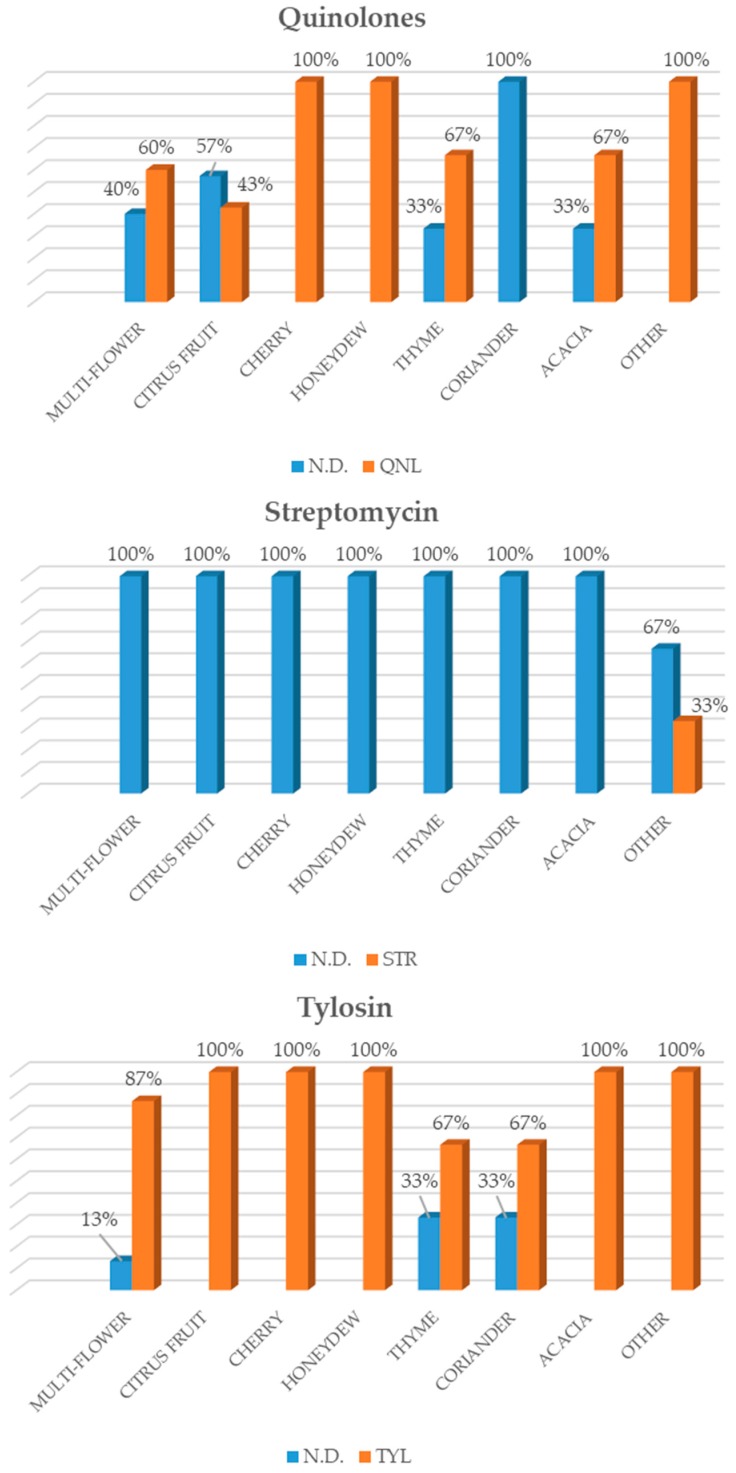

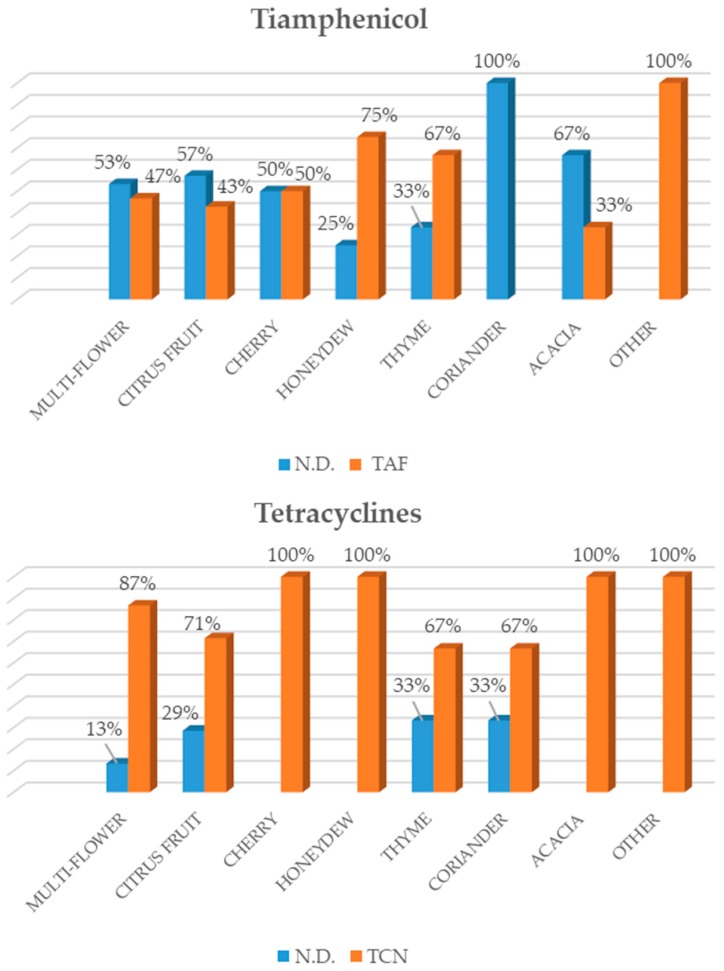
Percentage of positive samples and samples with a quantity of antibiotic undetectable by the instrument (N.D.), distinguished according to the antibiotic type. Figure 1 presents the second group of honey, consisting of forty-two samples produced in the Apulia region during 2017.

Among the sixty-six Apulian honeys analyzed, forty revealed the presence of antibiotics; conversely, twenty-six samples showed levels non-detectable by the Evidence Investigator™ Anti-Microbial array II, and consequently, they were indicated as N.D. (not detected). As can be observed in Figure 1, with regard to honey produced in 2017, of the six classes of antibiotics that we tested, tylosin (TYL) was detected in 38 honey samples, tetracyclines (TCN) in 36 honey samples, quinolones (QNL) in 27 honey samples, thiamphenicol (TAF) in 21, cephalosporins (CEFT) in 19, and finally, streptomycin (STR) was detected in just one honey sample. As regards the year of production, the results highlighted the different use of antibiotic substances between the two years that were monitored. In fact, of the 24 honey samples from 2016, only 2 showed positivity to quinolones, though in quantities slightly higher than the limits detectable by the instrument. On the other hand, of the 42 honey samples collected during 2017, 38 showed the presence of at least one of the six classes of antibiotics studied.

## 4. Discussion

According to the Codex Alimentarius (1981) [20], which is used in the EU, honey is a natural, sweet substance produced by honey bees to which no substance may be added, and from which none may be extracted. Therefore, the European Parliament [21], has called for continuous checks to be carried out on the quality of honey imported from third-party countries where legislation permits the treatment of bee colonies with antibiotics.

In recent years, several studies [4,14,22] have shown that chemical residues from veterinary drugs such as streptomycin, chloramphenicol, macrolides, sulphonamides, tetracyclines, quinolones and aminoglycosides and other contaminants such as pesticides and heavy metals have been found in honey, leading to concerns for the population.

Baggio et al. (2009) [23], highlighted that sulphonamides were the most widely used substances in honey in the Italian market, followed by tetracycline, streptomycin, tylosin and chloramphenicol. As regards the honey from local beekeepers in the Veneto Region (north-eastern Italy), a high number of samples tested positive for tylosin, as confirmed by our study. However, the results also showed a change with regard to the antibiotics applied to beehives over the last few years. In fact, besides the administration of traditional antibiotics such as sulphonamides and, in particular, tetracyclines, an increase in newer antibacterial substances such as streptomycin and tylosin was recorded among the active substances that were analysed [23].

Because low levels of antibiotics may favour the proliferation of antibiotic-resistant bacteria, there is growing interest in their presence and persistence in the environment [24]. The increasing concern about pharmaceuticals in honey has prompted scientific, industrial and regulatory bodies to assess their adverse effects on humans [11]. Previous studies [25,26] have demonstrated that veterinary medicines could persist in honey collected from hives several months after treatment. Indeed, according to Fussell et al. [27], ciprofloxacin was detected 283 days after treatment and this extended period indicated that the parent compound was a suitable marker for the detection of the use or abuse of ciprofloxacin in apiculture.

The results emerging from our work may have a dual explanation. First, is that beekeepers have had recourse to antibiotics to prevent and combat honeybee diseases. In fact, antibiotics have been used in beekeeping since 1940, as evidenced by several recent works [21,22,26]. The second hypothesis emerged from ISTAT (2017) [28] data on Apulian honey produced in 2017 which, due to the high temperatures and lack of rain, saw a loss in production of between 30–50%, depending on the different area of production. For example, the marked decrease in rainfall led to a reduction in the nectar sources available during the periods of normal honeybee foraging.

Table 2 shows mean monthly rainfall levels expressed in mm, highlighting that in the province of Bari average rainfall was much lower in all months of 2017, compared to the corresponding months of 2016, with peaks recorded in June (−85% mm of water) and July (−81% mm of water) compared to the previous year. The province of Taranto showed a decrease in the monthly average precipitation in all months, except for April, with peaks in March 2017 (−91% mm of water) and August (−90% mm of water) compared with the same months in 2016. Finally, the province of Foggia also recorded lower average rainfall, in all months except for May 2017, with minimum rainfall recorded in the months of July (−79% mm of water) and August (−90% mm of water) [28].

The results suggest that in some cases, the antibiotic is being used in a country belonging to the European community, such as Italy, even though the legislation clearly bans the use of antibiotics in beekeeping. In other cases, honey from non-EU countries (where the use of antibiotics in apiculture is allowed) were labelled as Apulian honey, thus constituting fraud on the part of the producer. Moreover, thanks to the innovative method used, we were able to analyse six different classes of antibiotics simultaneously.

The two limits of our study include the non-use of an official confirmatory method for the detection of antibiotic residues in honey, and, the non-use of pollen identification. While considering the limits of our study, it is clear that national controls should be further investigated because honey, which is generally considered a natural and healthy product [29] and always in great demand in the market, even as part of many sweet snacks for children, may become a risk for consumer health.

The “European Honey Breakfast” initiative launched in 2014 was open to all Member States and was a great success. The aim was to contribute to the education of children with regard to eating healthy food such as honey, and to promote the apiculture sector. However, particular attention should be paid to this food, and generally, to the apiculture sector in terms of agriculture, plant protection and sustainable farming as bees have a large impact on the ecological balance worldwide [20]. Moreover, the EU school fruit, vegetables and milk scheme programs allow Member States to include other local, regional or national specialties such as honey [20].

Considering that the EU Regulations place achieving “consumer health” in the food chain as a major objective, it is essential to ensure that the ban on the use of antibiotics in beekeeping practices is respected. The introduction of more controls would ensure not only the health of the consumer but also the health of the bees themselves, as well as environmental protection. In view of the results of this study, the need to monitor the presence of antibiotic molecules that are different from those contained in the NRCP is emphasized, and innovative screening methods were introduced that allowed us to obtain multi-residual data from food matrices.

## Figures and Tables

**Table 1:** Detection limits and assay range of the Evidence Investigator™ AM II analytes. Antibiotics with a value lower than the detection limits of the instrument cannot be detected and, consequently, they are indicated as N.D. (not detected).

Analyte	Honey LOD (ppb)	Assay Range (ppb)
Quinolones (QNL)	3.0	0–11.5
Ceftiofur (CEFT)	2.0	0–7.0
Thiamphenicol (TAF)	1.0	0–5.0
Streptomycin (STR)	5.0	0–75.0
Tylosin (TYL)	1.0	0–5.0
Tetracycline (TCN)	5.0	0–2.5

**Table 2:** Rainfall levels (average monthly rainfall, expressed in mm) detected by the monitoring network of the Apulia region stations in the provinces of Bari, Taranto and Foggia.

**Provinces**	**March**		**April**		**May**		**June**	
	**2016**	**2017**	**2016**	**2017**	**2016**	**2017**	**2016**	**2017**
Province of Bari	77.55	29.90	29.20	28.03	79.13	41.10	41.18	6.35
Province of Taranto	101.13	9.47	20.47	52.47	93.93	28.07	25.33	12.80
Province of Foggia	227.70	61.10	73.80	53.80	18.00	73.60	65.30	32.00
**Provinces**	**July**		**August**		**September**		**October**	
	**2016**	**2017**	**2016**	**2017**	**2016**	**2017**	**2016**	**2017**
Province of Bari	42.95	8.28	25.21	8.03	165.08	53.40	51.68	39.20
Province of Taranto	12.93	6.53	31.37	3.07	123.67	62.33	45.13	19.40
Province of Foggia	80.10	16.50	25.85	2.60	179.80	52.70	109.50	40.90
